# Unsuccessful Practitioners

**Published:** 1875-10

**Authors:** 


### Editorial.

### Unsuccessful Practitioners.

We copy elsewhere a reply to "Diploma," whose lament we published in our last month's issue. The letter was so suggestive in some of its details that we could not resist the temptation to copy it in full, notwithstanding its length; and the reply is so much to the point, in some respects, that we feel bound to give it also.

Starting with advantages possessed by but few young men, we must accept "Diploma's" qualifications as being above the average of young physicians. Having acquired his education and secured the charge of the surgical class at a large dispensary, he settles down in the metropolis and awaits practice. We may presume that the surgical class at the dispensary was secured to occupy spare time and afford opportunity for study and investigation. Of course, but few will be inclined for a moment to suppose that any idea of the advantages of this position as an advertising medium suggested itself to the mind of " Diploma." Yet, as he calls the Code of Ethics " absurd," we presume he did not object to his name appearing in the public prints in connection with the advertisement of said dispensary, as having charge of the *Surgical* deparment. Iu fact, we may presume that his own and the names of his colleagues were obligingly inserted in the local column occasionally, with their several departments specified, in order that the needy *poor* might not, through ignorance, suppose that Dr. A—— was devoted to Surgery, when that department was occupied by Dr. B——, Dr. A—— being in charge of the department of Gynæcology.

Sad to relate, " Diploma " did not succeed in the metropolis, and we can, in a measure, imagine his feelings when he gazed up the record of a year's work, charges against two patients, " from neither of whom had anything been collected." The lesson was doubtless a hard one, but it taught him that the crowded walks of city professional life contained no room, except in rare instances, for a beginner in the practice of medicine. A strange fascination, a wish for easy practice, or a desire to be near the centre of busy life, seems to draw young medical men into our large cities. In a few instances they gain a scant living for a few years, and gradually grow into a larger practice ; but for one who succeeds in city life there are two who fail and who seek practice in other and less crowded fields. If they choose right and are possessed of business faculties, which are necessary to success in any business, they succeed ; if not, they fail, and either seek other fields or fall into some other calling.

There seems to have been something lacking in " Diploma's " case. Although he was possessed of an excellent education, and was not disposed to coquette with his business, he easily looses that which he has gained in the new quarter to which he moves, and when a *rubbing* doctor and his wife step in to claim a portion of practice, he throws up his hands in despair because the leading minister in town patronizes the quack. Poor " Diploma " has spent so many of his years in colleges, hospitals and German laboratories, that he has failed to obtain that knowledge of the world and that insight into the character of the majority of its inhabitants, so necessary to successfully cope with the vanities and vexations of life. We could show him a town, not a hundred miles from where we are now sitting, whose leading men, aye, and intelligent ones, too, resort to just such " pestilent, vulgar, and ill-educated quacks " as those to whom he refers ; but the physicians are not discouraged thereby, but expect to treat those same people in due time, when the quacks have bled their purses to their satisfaction. Certainly, if the rubbing doctor cured the " valetudinarians of all the churches," " Diploma " has only himself to blame for not doing as well. The lamenting

Jeremiah is condemned out of his own mouth when he tells us that $400 represented payments from one-fifth of his patients, the remaining four-fifths being still his debtors, though most of them were fully able to pay him. If he conducts his mercantile business which he intends to seek on that principle, he will make a failure of that as well. If he had learned some of the business capacity of his neighbor, who could not read his own diploma, but who doubtless succeeded in getting practice, the sad lament would never have been uttered ; he would have left the crowded "Beeler's quarters," and sought some country village where the call was urgent for a good doctor—one who knew his business (and many such places exist)—and there, although the society might not have been as congenial, nor the practice as easy, success would have attended his earnest, well directed endeavor.

We cannot but admit that in the crowd of young men who are annually sent out from our medical colleges many incompetent physicians exist, but we are not disposed to view the matter in as dark a light as "Diploma" places it, not to impute the sordid motives to the teachers in our medical schools that he charges them with, and the many important and valuable contributions which American physicians have made to medical science seems to contradict the assertion which is so often made by jealous or disappointed men, that American medical education is a farce. If the very men who cry out against the system, and who, like the Pharisees, thank God that they are not as other men are, were to lament less at the exceeding weakness of the system, and do more to build up its strong points, there would be less cause for complaint. But as long as College Faculties exist, so long will there be men in the ranks of the profession envious of the supposed advantages attending membership of those bodies.

